# Evaluation of *In Vitro* Antioxidant Properties of Methanol and Aqueous Extracts of *Parkinsonia aculeata* L. Leaves

**DOI:** 10.1155/2013/604865

**Published:** 2013-11-14

**Authors:** Sonia Sharma, Adarsh Pal Vig

**Affiliations:** Department of Botanical and Environmental Sciences, Guru Nanak Dev University, Amritsar, Punjab 143005, India

## Abstract

In the present study, methanol and aqueous extracts of *Parkinsonia aculeata* L. leaves were prepared and analyzed for phytochemical analysis and antioxidant potential in different *in vitro* assays. Antioxidant activity was studied using DPPH, CUPRAC, reducing power assay, deoxyribose degradation (site and nonsite specific), ferric reducing antioxidant potential (FRAP), ferric thiocyanate (FTC), thiobarbituric acid (TBA), and molybdate ion reduction, respectively. The total phenolic contents of the methanol and aqueous leaf extract were 39 mg GAE/g and 38 mg GAE/g, whereas flavonoid contents of these extracts were found to be 0.013 mg RE/g and 0.006 mg RE/g, respectively. From the two extracts, the methanol extract shows maximum inhibition (%) of 57.82%, 71.23%, 48.26%, 69.85%, and 52.78% in DPPH, nonsite- and site-specific, FTC, and TBA assays and absorbance of 0.669 and 0.241 in reducing power and CUPRAC assays at the highest concentration tested. UPLC analysis was done to determine the presence of various types of polyphenols present in plant extracts.

## 1. Introduction

Biological combustion involved in various processes produces harmful products or intermediates called reactive oxygen species or free radicals. Excess of free radicals in living beings has been known to cause various problems like asthma, cancer, cardiovascular diseases, liver diseases, muscular degeneration, and other inflammatory processes [1], resulting in the so-called oxidative stress. Oxidative stress is defined as imbalance between oxidants and antioxidants and causes damage in all types of biomolecules like protein, nucleic acid, DNA, and RNA [2]. Hence, the balance between reactive species or free radicals and antioxidants is believed to be a critical concept for maintaining a good biological system. Antioxidants act as free radical scavengers, reducing agents, quenchers of singlet oxygen molecule, and activators for antioxidative enzyme to suppress the damage induced by free radicals in biological system.

It has been found by many researchers that there is an inverse association between the mortality from age-related diseases and the consumption of plant products [3], which could be due to the presence of various antioxidant compounds, especially, phenolics, which are the most reactive compounds. Antioxidants present in plant products help in the stimulation of cellular defence system and biological system against oxidative damage. *Parkinsonia aculeata* (*P. aculeata*) is small spiny deciduous tree, native to tropical America, and, now, it has been introduced and well cultivated in South Africa, Israel, Uganda, and India [4, 5]. It is traditionally described to treat fever and malaria and as an abortifacient [6]. The leaves of this plant are prescribed to treat rheumatism [7]. However, information pertaining to the antioxidant properties of *P. aculeata* is meager. However, in this study, the antioxidant activity of this plant was investigated in detail by employing many different *in vitro* antioxidant assays.

## 2. Materials and Methods

### 2.1. Plant Material and Extraction Procedure

The leaves of *P. aculeata* were collected near Guru Nanak Dev University (Punjab, India). Botanical identification was made at the Herbarium of the Department of Botanical and Environmental Sciences, GNDU, where a voucher of specimen (Accession no. 6774, dated: 17 June 2012) was deposited. The plant sample was ground to fine powder, and the precisely weighed amount of the powder was extracted with 80% methanol and aqueous solvents and was vacuum dried with Buchi Rotavapor to obtain the dried methanol and aqueous extracts. These extracts were used for the phytochemical analysis and determination of antioxidant activities and total phenolic and flavonoid contents.

### 2.2. Chemicals

Folin-Ciocalteu reagent, sodium carbonate, gallic acid, rutin, aluminium chloride, sodium nitrate, sodium hydroxide, 2,2-diphenyl-1-picrylhydrazyl (DPPH), trichloroacetic acid, potassium ferricyanide, sodium acetate buffer, neocuproine, deoxyribose, EDTA, potassium phosphate buffer, hydrogen peroxide, ascorbic acid, TBA, 2,4,6tripyridyl-s-triazine (TPTZ), ferric chloride, HCl, ammonium molybdate, sodium phosphate, sulfuric acid, ammonium thiocyanate, and all other chemicals used were of analytical grade.

### 2.3. Total Phenolic Determination

Total phenolic content was determined using the Folin-Ciocalteu reagent [8]. 0.1 mL of extract was diluted with 1 mL distilled water and added to solution of 0.5 mL of Folin-Ciocalteu reagent and 1.5 mL of 20% sodium carbonate solution. The reaction mixture was incubated for 2 hours, and, finally, the volume was raised to 10 mL, and the absorbance was read at 765 nm. Gallic acid (0–200 *μ*g/mL) was used for calibration of standard curve. The total phenolic content was expressed as milligram gallic acid equivalent (mg GAE)/g dry weight of plant material.

### 2.4. Total Flavonoid Determination

The method given by Zhishen et al. [9] was used for analyzing total flavonoid content (TFC) employing rutin as a standard. To 1 mL of extract, solution of distilled water (dH_2_O), 5% NaNO_2_, and 10% AlCl_3_ were added and incubated for 5 min. To the above mixture, 1 M NaOH and 2.4 mL dH_2_O were added to get the final volume of 10 mL. The absorbance of samples was taken at 510 nm by US-VIS spectrophotometer. The total flavonoid content was expressed as mg rutin equivalents per gram (mg RE/g) through the calibration curve.

### 2.5. DPPH Free Radical Scavenging Activity

The hydrogen atom donating ability of the different plant extracts was determined by the decolorization of methanol solution of 2,2-diphenyl-1-picrylhydrazyl (DPPH) [10]. 0.2 mL of extract was added to 3 mL of 0.1 mM DPPH solution, and absorbance was read at 517 nm. The decrease in absorption was correlated with the percent inhibition of samples. The percentage of inhibition was calculated by the following:

(1) $$\begin{array}{c} \%\;\text{antioxidant}\;\text{activity}=\left[\frac{\text{Ac}-\text{As}}{\text{Ac}}\right]\times 100, \end{array}$$

where Ac = absorbance of control and As = absorbance of sample.

### 2.6. Reducing Power Assay

The reducing power of the extracts of *P. aculeate * L. leaves was determined according to the method of Oyaizu [11]. 1 mL of extract was added with 2.5 mL of phosphate buffer and 2.5 mL of 1% potassium ferricyanide. The reaction mixture was incubated for 20 minutes at 50°C, and, after that, 2.5 mL of 10% TCA was added and centrifuged. The supernatant was mixed with 2.5 mL of distilled water and 0.5 mL of FeCl_3_, and the absorbance was read at 700 nm. The assay was carried out in triplicate, and the results are expressed as mean ± standard error (SE). Increase in absorbance of sample with concentrations indicates high reducing potential of the samples.

### 2.7. Cupric Ions Reducing Assay (CUPRAC)

In order to determine the cupric ions (Cu^2+^) reducing ability of methanol and aqueous extracts of *P. aculeate * L. leaves, the method proposed by Apak et al. [12] was used. In this assay, 0.01 M of CuCl_2_ solution, 7.5 mM of ethanol neocuproine solution, and 1.0 M of CH_3_COONH_4_ buffer solution were added to each test tube containing different concentrations of standard antioxidant (gallic acid) or extracts, respectively. Finally, total volume was adjusted to 2 mL with dH_2_O and incubated for 30 minutes at room temperature. Absorbance was measured at 450 nm against a reagent blank. Increased absorbance of the reaction mixture shows increased reduction capability of solution.

### 2.8. Nonsite-Specific Hydroxyl Radical Scavenging Activity

Nonsite-specific hydroxyl radical scavenging activity of extracts was measured according to the method of Aruoma et al. [13]. For this assay, 1 mL of Haber-Weiss reaction mixture (2-deoxyribose, Fe(III) chloride, EDTA, and H_2_O_2_) was added with plant extract, and the reaction was started by adding ascorbic acid and incubated for 1 hour at 37°C. After incubation time, 1 mL of the above solution, 1 mL of TBA, and 1mL of TCA were added, and the mixture was heated for 90 minutes. The pink color development was measured at 532 nm against a blank containing phosphate buffer.

### 2.9. Site-Specific Hydroxyl Radical Scavenging Activity

This procedure is similar to that used to measure the nonsite-specific hydroxyl scavenging activity. In this assay, EDTA was replaced by potassium phosphate buffer [14].

The inhibitory effect of sample was calculated as follows:

(2) %  hydroxyl  radical  scavenging  capacity=(1−AsAc)×100.

Here, Ac = absorbance of control, and As = absorbance of sample solution.

### 2.10. Ferric Reducing Antioxidant Power (FRAP)

Reducing power of the two extracts (methanol and aqueous) of *P. aculeata* was done according to Benzie and Strain [15] with some modifications. Readings of the colored product (ferrous tripyridyltriazine complex) were then measured at 593 nm. The standard curve was linear between 100 and 1000 *μ*M FeSO_4_. Results are expressed in *μ*M (Fe(II)/g) dry mass [16]. Decreased absorbance indicates ferric reducing power capability of sample [17].

### 2.11. Total Antioxidant Capacity (Phosphomolybdic Acid Method)

The antioxidant activity of methanol and aqueous extracts was evaluated by the transformation of Mo(VI) to Mo(V) to form phosphomolybdenum complex [18]. In this assay, 0.3 mL of extract was incubated with reaction mixture (0.6 M sulfuric acid, 28 mM sodium phosphate, and 4 mM ammonium molybdate) for 90 minutes, and absorbance was read at 695 nm. The results were expressed as AAE/100 mg dry weight of extract.

### 2.12. Ferric Thiocyanate (FTC) Method

The standard method described by Kikuzaki et al. [19] was used for ferric thiocyanate determination. 4 mg of extract dissolved in 4 mL of 99.5% ethanol, 4.1 mL of 2.51% linoleic acid in 99.5% ethanol, 8.0 mL of 0.02 M phosphate buffer (pH 7.0), and 3.9 mL of distilled water contained in covered test tubes was placed in an oven at 40°C. 0.1 mL of the reaction mixture from the above solution was transferred to a test tube, and 9.7 mL of 75% aqueous ethanol followed by 0.1 mL of 30% aqueous ammonium thiocyanate and 0.1 mL of 0.02 M ferrous chloride in 3.5% hydrochloric acid were added. The absorbance of the resulting mixture of red color was measured at 500 nm after every 24 hours until the absorbance reached its maximum value. Gallic acid was used as positive control, while the negative control used was the mixture without the plant extract.

### 2.13. Thiobarbituric Acid (TBA) Method

The method of Kikuzaki and Nakatani [20] was used for the determination of free radicals present in the methanol and aqueous leaf extracts. In this assay, 2 mL of 20% trichloroacetic acid and 2 mL of 0.67% of thiobarbituric acid were added to 1 mL of sample solution of plant extract from the FTC method. The mixture was placed for 10 min in a water bath and then centrifuged after cooling at 3000 rpm. The absorbance activity of the supernatant was measured at 552 nm and recorded after it has reached its maximum value.

### 2.14. Statistical Analysis

Each experiment was performed at least three times, and results were recorded as mean ± standard error (SE).

### 2.15. Ultra Performance Liquid Chromatography (UPLC)

Methanol and aqueous extracts of *P. aculeate * L. were subjected to UPLC in order to identify the presence of various polyphenolic compounds like gallic acid, epicatechin, umbelliferone, coumaric acid, and so forth. For UPLC analysis, dried leaves powder was extracted with 80% methanol and aqueous solvents. The supernatants were collected and dried on rotary evaporator. The dried extracts were dissolved in methanol (HPLC grade) and analyzed for the presence of different polyphenols.

## 3. Results and Discussion

It was reported that phenolic compounds were associated with antioxidant activity and that they play an important role in stabilizing lipid peroxidation [21]. The total phenolic contents of methanol and aqueous leaf extracts were 39 mg GAE/g and 38 mg GAE/g (*y* = 0.001*x* + 0.034; *R* ^2^ = 0.990). The total flavonoid contents of methanol and aqueous extracts of leaves of this plant were found to be 0.013 mg RE/g and 0.006 mg RE/g, respectively, with reference to standard curve. These phytochemical compounds are known to provide support for bioactive properties of plant, and thus they are responsible for the antioxidant properties of *P. aculeata*. L. 

**Figure 1:**
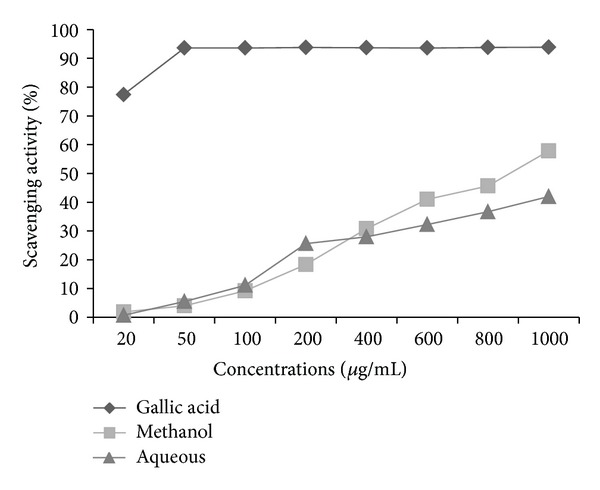
DPPH radical scavenging activity of methanol and aqueous extracts of *P. aculeata* L. leaves (values are average of triplicate experiment and are represented as mean ± SE).

**Figure 2:**
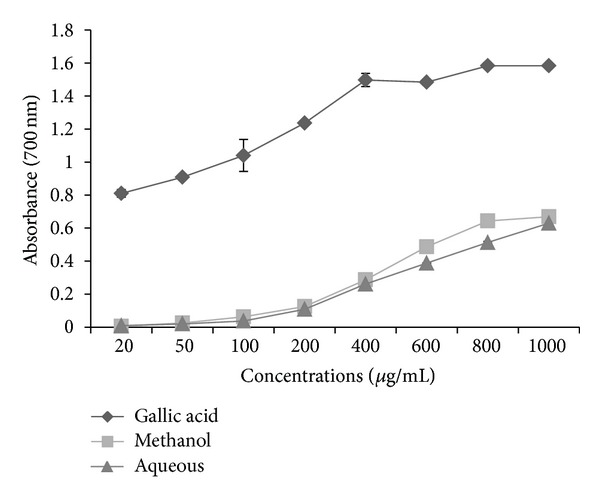
Reducing power of extracts of *P. aculeata* L. leaves (values are average of triplicate experiment and are represented as mean ± SE).

**Figure 3:**
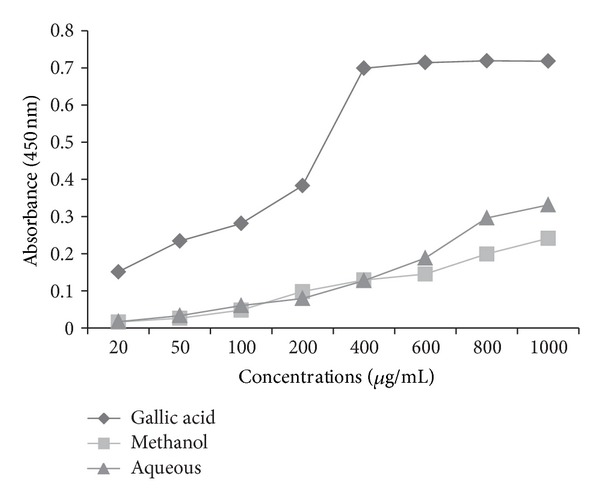
Antioxidant activity of different extracts of *P. aculeata* L. leaves and standard (gallic acid) by using CUPRAC assay.

The *in vitro* antioxidant assay performed on this plant reveals significant antioxidant potential compared with gallic acid as a standard. DPPH radicals are widely used in the model system to investigate the scavenging activity of several natural phytocompounds. The result of DPPH scavenging activity in this study indicates that the plant was potentially active. Methanol extract shows % age inhibition of 57.82 as compared to aqueous extract which shows 41.97% age inhibition at the highest concentration of 1000 *μ*g/mL (Figure 1). The DPPH contains an odd electron, which is responsible for purple color, and absorbance wavelength of 517 nm [22]. The methanol and aqueous extracts of *P. aculeate * L. were estimated using potassium ferric cyanide reduction method. In this assay, the yellow color formed in the reaction is significant indicator of antioxidant activity. From the two extract, methanol extract shows high absorbance of 0.669, then comes the absorbance of aqueous extract that is 0.63 at the highest concentration (Figure 2). In the CUPRAC assay, Cu(II)-Nc which is the main oxidizing agent gets reduced to colored Cu(I)-Nc chelate which shows maximum absorbance at 450 nm.  Figure 3 shows the maximum absorbance of 0.241 and 0.331 of methanolic and aqueous extract at higher concentration, whereas standard, that is, gallic acid, shows absorbance of 0.718 at the same concentration. In this assay, a higher absorbance indicates higher antioxidant activity. Singh et al. [23] studied antioxidant properties using DPPH assay of leaves extracts, that is, methanol, chloroform, ethyl acetate, and aqueousness of *P. aculeate * L., and found that different phytochemicals, present in the leaves, are responsible for the high antioxidant potential.

**Figure 4:**
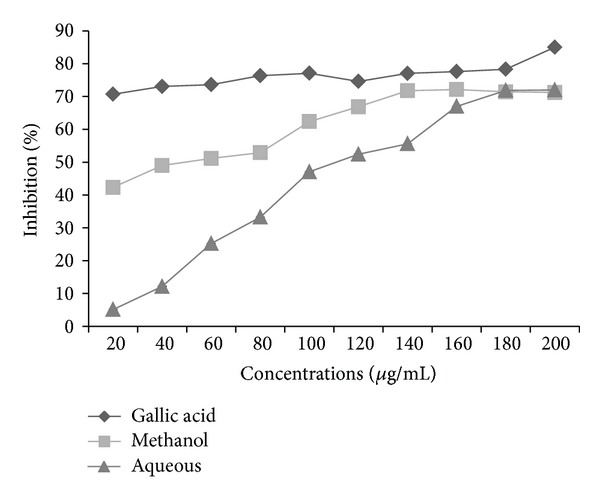
Hydroxyl radical scavenging activity (-EDTA) of *P. aculeata* L. and gallic acid (results are represented as mean ± SE of triplicate experiment).

**Figure 5:**
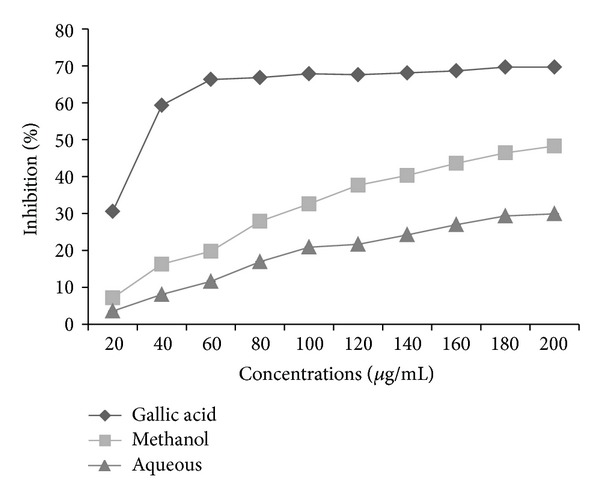
Hydroxyl radical scavenging activity of leaves extracts of *P. aculeata* L. and gallic acid (results are represented mean ± SE of triplicate experiment).

The extracts were assessed for their radical scavenging potential using site-specific and nonsite-specific deoxyribose degradation assay. In nonsite specific degradation assay, methanol and aqueous extracts show the inhibition of 71.232 and 72.019% at the same concentration (Figure 4). In the site-specific assay, methanol extract showed 48.268% inhibition, whereas aqueous extract shows inhibition of 29.921% at 200 *μ*g/mL (Figure 5). The standard (gallic acid) shows the inhibition of 69.68 and 85.005% in site- and nonsite-specific degradation assay at the 200 *μ*g/mL. These results show the potent antioxidant nature of different extracts of *P. aculeata *L. The antioxidant compounds are responsible for the reduction of ferric (Fe^3+^) form to ferrous (Fe^2+^) form. The addition of FeCl_3_ to the ferrous form led to the formation of blue colored complex. So the reduction ability can be determined by measuring the colored complex at 700 nm [24]. The FRAP values of methanol and aqueous extracts were found to be 498 (*μ*M Fe(II)/g) and 461 (*μ*M Fe(II)/g) from the standard curve obtained from the FeSO_4_7H_2_O.

**Figure 6:**
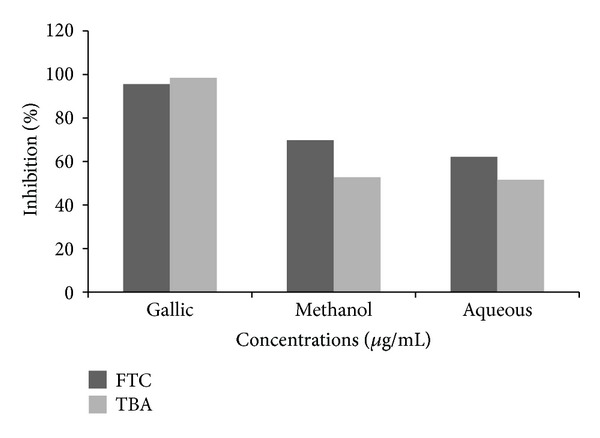
Antioxidant properties of leaves extracts of *P. aculeate * L. and gallic acid, determined by FTC and TBA methods. FTC: ferric thiocyanate; TBA: thiobarbituric acid.

**Figure 7:**
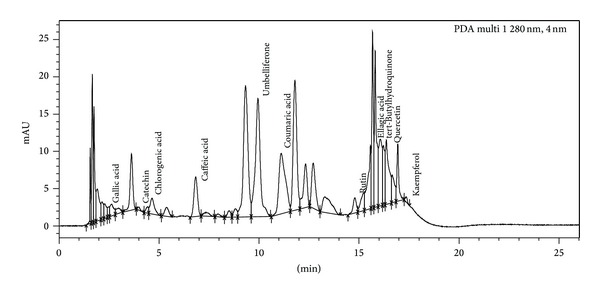
UPLC analysis of methanol extract of leaves of *P. aculeata* L.

**Figure 8:**
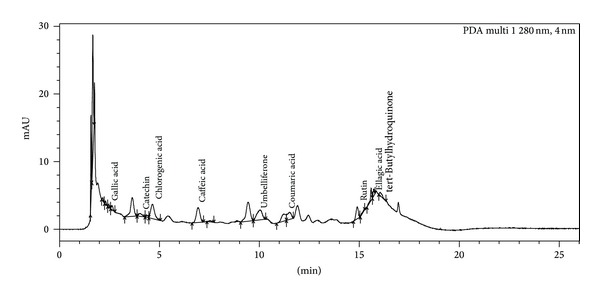
Chromatogram showing the presence of various types of polyphenols in the aqueous extract of *P. aculeata* L.

 The results obtained from phosphomolybdic acid assay show that methanol extract has strong ability to reduce Mo(VI) to Mo(V) by donating electron. This evaluated that both methanol and aqueous extracts show reduction ability of 20.75 mg AAE/100 mg and 8 mg AAE/100 mg dry weight of extract. These values were calculated from the regression equation obtained for ascorbic acid as a standard curve. The antioxidant activity of methanol and aqueous extracts was also determined by using ferric thiocyanate (FTC) and thiobarbituric acid (TBA). The FTC method was used to measure peroxide amount at the starting phase of peroxidation, whereas TBA method was used to measure the concentration of free radicals present at the end of peroxide oxidation [25] on the sixth day of experiment. Methanol and aqueous extracts show FTC values of 69.85% and 62.12%, whereas, in TBA method, methanol and aqueous extracts show 52.78% and 51.56% inhibition at the highest concentration (1000 *μ*g/mL) (Figure 6). Standard, that is, gallic acid, shows the FTC and TBA values of 95.54% and 98.46% at the same concentration on the sixth day. It was found from the UPLC analysis of extracts that various types of polyphenols are present in the crude extracts, and those are maybe responsible for the antioxidant properties of this plant. On the basis of chromatogram of methanol extract, it was found that leaves contain various types of polyphenols like gallic acid, catechin, chlorogenic acid, epicatechin, tert-Butylhydroquinone, and so forth. (Figure 7), and chromatogram of aqueous extract (Figure 8) also showed the presence of various polyphenols like gallic acid, catechin, chlorogenic acid, caffeic acid, ellagic acid, tert-Butylhydroquinone, and so forth. Gupta et al. [26] also observed pharmacognostic characters which include morphology, T.S, powder microscopy, physicochemical characteristics, and phytochemical screening from alcoholic and aqueous extracts of the bark of *Parkinsonia aculeata *L.

This study affirms the *in vitro* antioxidant potential of methanol and aqueous extracts of leaves of *P. aculeata*. L. Results are compared with values of different standards such as gallic acid and ascorbic acid. To our knowledge, this is the first report demonstrating that methanol and aqueous extracts of *P. aculeate * L. have antioxidant activity as seen in the DPPH, free radical assay, CUPRAC, site- and nonsite-specific hydroxyl radical scavenging assay, FRAP, TAC, FTC, and TBA assay. From the two extracts, methanol extract shows high antioxidant properties than aqueous extract. But further studies are required to clarify the *in vivo* potential of this plant.

## Supplementary Material

Supplementary Material: Parkinsonia aculeata L. (P. aculeata) is small spiny deciduous tree, native to tropical America, and introduced and well cultivated in South Africa, Israel, Uganda and India. Antioxidant potential of P. aculeata is found to be due to the presence of different phytochemicals, present in the leaves. On the basis of chromatogram of leaves extract, it was found that leaves contain various types of polyphenols like gallic acid, catechin, chlorogenic acid, epicatechin, tert-Butyl hydroquinone, caffeic acid, Ellagic acid, isoorientin, orientin and tert-Butyl hydroquinone etc.

## References

[1] Sen S, Chakraborty R, Sridhar C, Reddy YSR, De B (2010). Free radicals, antioxidants, diseases and phytomedicines: current status and future prospect. *International Journal of Pharmaceutical Sciences Review and Research*.

[2] Dröge W (2002). Free radicals in the physiological control of cell function. *Physiological Reviews*.

[3] Sharma S, Nagpal A, Vig AP (2012). Genoprotective potential of *Brassica juncea* (L.) Czern. against mercury-induced genotoxicity in A*llium cepa* L. *Turkish Journal of Biology*.

[4] Wagner WL, Herbst DR, Sohmer SH (1999). *Manual of the Flowering Plants of Hiwaii*.

[5] Pier (2000). *Invasive Plant Species: Parkinsonia Aculeata*.

[6] Divya B, Mruthunjaya K, Manjula SN (2011). Parkinsonia aculeata: a Phytopharmacological review. *Asian Journal of Plant Sciences*.

[7] Orwa C, Mutua A, Kindt R, Jamnadass R, Simons A Agroforestree database: a tree reference and selection guide version 4.0. Nairobi, Kenya. http://eoispecies.Lifedesks.Org/node/3416.

[8] Singleton VS, Rossi JA (1965). Colorimetric of total phenolics with phosphomolybdic- phosphotungstic acid reagents. *American Journal of Enology and Viticulture*.

[9] Zhishen J, Mengcheng T, Jianming W (1999). The determination of flavonoid contents in mulberry and their scavenging effects on superoxide radicals. *Food Chemistry*.

[10] Blois MS (1958). Antioxidant determinations by the use of a stable free radical. *Nature*.

[11] Oyaizu M (1986). Studies on product of browning reaction prepared from glucose amine. *Journal of Nutrition*.

[12] Apak R, Güçlü K, Özyürek M, Karademir SE (2004). Novel total antioxidant capacity index for dietary polyphenols and vitamins C and E, using their cupric ion reducing capability in the presence of neocuproine: CUPRAC method. *Journal of Agricultural and Food Chemistry*.

[13] Aruoma OI, Grootveld M, Halliwell B (1987). The role of iron in ascorbate-dependent deoxyribose degradation. Evidence consistent with a site-specific hydroxyl radical generation caused by iron ions bound to the deoxyribose molecule. *Journal of Inorganic Biochemistry*.

[14] Shon M-Y, Kim T-H, Sung N-J (2003). Antioxidants and free radical scavenging activity of *Phellinus baumii* (*Phellinus of Hymenochaetaceae*) extracts. *Food Chemistry*.

[15] Benzie IFF, Strain JJ (1996). The ferric reducing ability of plasma (FRAP) as a measure of “antioxidant power”: the FRAP assay. *Analytical Biochemistry*.

[16] Shajiselvin CD, Kottai Muthu A (2010). In-vitro antioxidant studies of various extracts of whole plant of *Borreria hispida* (Linn). *The Research Journal of Pharmaceutical, Biological and Chemical Sciences*.

[17] Koksal E, Bursal E, Dikici E, Tozoglu F, Gulcin I (2011). Antioxidant activity of Melissa officinalis leaves. *Journal of Medicinal Plant Research*.

[18] Prieto P, Pineda M, Aguilar M (1999). Spectrophotometric quantitation of antioxidant capacity through the formation of a phosphomolybdenum complex: specific application to the determination of vitamin E. *Analytical Biochemistry*.

[19] Kikuzaki H, Usuguchi J, Nakatani N (1991). Constituents of Zingiberaceae. I. Diarylheptanoids from the rhizomes of ginger (*Zingiber officinale* roscoe). *Chemical and Pharmaceutical Bulletin*.

[20] Kikuzaki H, Nakatani N (1993). Antioxidant effect of some ginger constituents. *Journal of Food Science*.

[21] Vijayabaskar P, Shiyamala V (2012). Antioxidant properties of seaweed polyphenol from *Turbinaria ornate* (Turner) J. Agardh, 1848. *Asian Pacific Journal of Tropical Biomedicine*.

[22] Ferreira ICFR, Baptista P, Vilas-Boas M, Barros L (2007). Free-radical scavenging capacity and reducing power of wild edible mushrooms from northeast Portugal: individual cap and stipe activity. *Food Chemistry*.

[23] Singh P, Shrivastava R, Saxena RC, Sharma M, Karchuli MS, Tripathi J (2011). Phytochemical screening and evaluation of antioxidant activity of *Parkinsonia aculeate* L. (Family-Leguminoseae) leaves extract. *International Journal of PharmTech Research*.

[24] Chung Y-C, Chang C-T, Chao W-W, Lin C-F, Chou S-T (2002). Antioxidative activity and safety of the 50 ethanolic extract from red bean fermented by *Bacillus subtilis* IMR-NK1. *Journal of Agricultural and Food Chemistry*.

[25] Aiyegoro OA, Okoh AI (2010). Preliminary phytochemical screening and In vitro antioxidant activities of the aqueous extract of Helichrysum longifolium DC. *BMC Complementary and Alternative Medicine*.

[26] Gupta MK, Kenganora M, Banerjee A, Saini L, Kumar V (2011). Pharmacognostical and phytochemical evaluation on the bark of *Parkinsonia aculeata* Linn. *Journal of Pharmaceutical Science and Bioscientific Research*.

