# Supplementary figures and images for: Evaluation of In Vitro Antioxidant Properties of Methanol and Aqueous Extracts of Parkinsonia aculeata L. Leaves
Source: ScientificWorldJournal. 2013 Nov 14;2013:604865. doi: 10.1155/2013/604865 (PMC3848342; doi:10.1155/2013/604865)

[
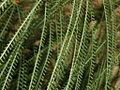
](http://en.wikipedia.org/wiki/File:Caesalpiniaceae_-_Parkinsonia_aculeata-2.JPG)

Supplement: Supplementary file 1 — Supplementary Material: Parkinsonia aculeata L. (P. aculeata) is small spiny deciduous tree, native to tropical America, and introduced and well cultivated in South Africa, Israel, Uganda and India. Antioxidant potential of P. aculeata is found to be due to the presence of different phytochemicals, present in the leaves. On the basis of chromatogram of leaves extract, it was found that leaves contain various types of polyphenols like gallic acid, catechin, chlorogenic acid, epicatechin, tert-Butyl hydroquinone, caffeic acid, Ellagic acid, isoorientin, orientin and tert-Butyl hydroquinone etc. [file 604865.f1.doc]
